# Influence of Co_3_O_4_ Nanostructure Morphology on the Catalytic Degradation of p-Nitrophenol

**DOI:** 10.3390/molecules28217396

**Published:** 2023-11-02

**Authors:** Huihui Chen, Mei Yang, Yuan Liu, Jun Yue, Guangwen Chen

**Affiliations:** 1 School of Chemistry and Chemical Engineering, Henan University of Technology, Zhengzhou 450001, China; chenhuihui@haut.edu.cn (H.C.); liuyuan2020@haut.edu.cn (Y.L.); 2 Dalian Institute of Chemical Physics, Chinese Academy of Sciences, Dalian 116023, China; yangmei@dicp.ac.cn; 3 Department of Chemical Engineering, Engineering and Technology Institute Groningen, University of Groningen, 9747 AG Groningen, The Netherlands

**Keywords:** cobalt oxide, morphology control, p-nitrophenol, nanostructure, catalysis

## Abstract

The design and fabrication of nanomaterials with controllable morphology and size is of critical importance to achieve excellent catalytic performance in heterogeneous catalysis. In this work, cobalt oxide (Co_3_O_4_) nanostructures with different morphologies (nanoplates, microflowers, nanorods and nanocubes) were successfully constructed in order to establish the morphology–property–performance relationship of the catalysts. The morphology and structure of the nanostructured Co_3_O_4_ were characterized by various techniques, and the catalytic performance of the as-prepared nanostructures was studied by monitoring the reduction of p-nitrophenol to p-aminophenol in the presence of excess NaBH_4_. The catalytic performance was found to be strongly dependent on their morphologies. The experimental results show that the pseudo-first-order reaction rate constants for Co_3_O_4_ nanostructures with various shapes are, respectively, 1.49 min^−1^ (nanoplates), 1.40 min^−1^ (microflowers), 0.78 min^−1^ (nanorods) and 0.23 min^−1^ (nanocubes). The Co_3_O_4_ nanoplates exhibited the highest catalytic activity among the four nanostructures, due to their largest specific surface area, relatively high total pore volume, best redox properties and abundance of defect sites. The established correlation between morphology, property and catalytic performance in this work will offer valuable insight into the design and application of nanostructured Co_3_O_4_ as a potential non-noble metal catalyst for p-nitrophenol reduction.

## 1. Introduction

Nanomaterials have been overwhelmingly developed in the fields of electronics, optics, biology, medicine, environmental science and catalysis in recent decades, due to their unique physical, chemical and biological properties that differ from those of bulk materials [1]. Among these, transition metal oxide nanomaterials have attracted particular research interest for potential applications in catalysis because of their abundant reserves, low cost, high stability and strong redox properties. However, the catalytic activity of transition metal oxides needs to be further improved compared with the excellent performance of noble metal catalysts [2]. Various strategies have been adopted to improve the catalytic performance of transition metal oxides, such as size control [3], crystal plane or morphology modulation [4], element doping, surface defect fabrication [5], pore introduction [6] and compound recombination [7]. For example, atomically dispersed Co catalysts anchored in porous nitrogen-doped carbon (Co_SA_-N-C) showed outstanding performance in removing low concentrations of HCHO at room temperature [8]. The catalytic activity of Ce-doped CuMn_8_O_x_ (2.5% Ce; molar percentage) was found to be higher than that of CuMn_8_O_x_ for the oxidation of CO at a lower temperature (65 °C) [9]. Due to the synergistic effect of ZIF-8 and TiO_2_, the ZIF-8@TiO_2_ micron composite has been used as a highly efficient photocatalyst for the degradation of tetracycline [10]. Therefore, with the rapid development of nanotechnology and the use of various regulation methods, transition metal oxides as catalysts are expected to have potential in partially substituting noble metal catalysts.

As a typical transition metal oxide, Co_3_O_4_ has been extensively used as a heterogeneous catalyst in many important chemical processes, such as low temperature oxidation of CO [11], CH_4_ combustion [12], oxygen evolution reaction [13], decomposition of H_2_O_2_ [14], oxidation of ethylene [15] and reduction of p-nitrophenol (p-NP) [16]. In general, the physicochemical properties of Co_3_O_4_ are significantly influenced by its morphology [17]; thus, it is of great significance to develop suitable preparation methods to obtain Co_3_O_4_ nanomaterials with controllable morphology and size in order to upgrade the properties of Co_3_O_4_ and further improve its catalytic performance. To date, many Co_3_O_4_ nanomaterials with different shapes have been successfully fabricated. For example, Yan et al. [18] synthesized Co_3_O_4_ nanoflower clusters by a simple low-temperature hydrothermal method. The catalytic activity of the as-prepared Co_3_O_4_ nanoflower clusters for the degradation of gaseous toluene appeared to be far superior to that of Co_3_O_4_ blocks under the same reaction conditions. Xie et al. [19] reported the synthesis of Co_3_O_4_ nanorods by a precipitation–calcination method. The catalyst exhibited surprisingly high catalytic activity for CO oxidation at temperatures as low as −77 °C. Ultrathin Co_3_O_4_ nanosheets were also synthesized by a facile solvothermal method for the catalytic oxidation of formaldehyde at room temperature [20].

p-Nitrophenol (p-NP), which is toxic, mutagenic and non-biodegradable, is one of the most common organic pollutants in industrial and agricultural wastewater. Due to its ability to bioaccumulate and its stability, effluents containing p-NP can cause serious contamination to soil and groundwater if discharged directly into the environment without treatment [21]. In addition, its accumulation and residue in the environment may irritate eyes and skin, damage the liver, kidney and nervous system, and even cause chromosomal aberrations or blood disorders in humans [22]. Many strategies have been proposed for the degradation of p-NP, such as adsorption [23], microbial degradation [24], photocatalytic degradation [25], electrochemical methods [26] and chemical reduction [27]. Compared with other techniques, the catalytic reduction of p-NP to p-aminophenol (p-AP) by an excess NaBH_4_ aqueous solution is one of the most feasible and simple methods due to its high efficiency and facile operating conditions [28]. Moreover, the only reduction product, p-AP, is not only of low toxicity, but also an important intermediate in the production of various pharmaceuticals, agrochemicals, dyes, chelating agents and photographic developers [29].

The transition metal oxides, such as Co_3_O_4_, CuO, Fe_2_O_3_ and NiO, were found to be catalytically active for the reduction of p-NP to p-AP [30,31]. In addition, several studies have shown that the shape and size of the catalyst have a significant effect on the catalytic activity for the reduction of p-NP. For example, Konar et al. [32] synthesized rod-, spherical-, star-, and flower-shaped CuO nanostructures. The star-shaped CuO showed the maximum catalytic activity for the reduction of p-NP in the presence of NaBH_4_, which is correlated to their higher specific surface area, positive surface charge and the presence of a high index facet. Che et al. [33] obtained leaf-like, dumbbell-like, and flower-like CuO nanostructures via a simple oil bath route by introducing different additives. The order of catalytic activity for the reduction of p-NP to p-AP is leaf-like nanosheets > dumbbell-like architectures > flower-like nanostructures. In contrast, there are only a few reports available in the literature showing the effect of Co_3_O_4_ morphology on the reduction of p-NP. For example, conical Co_3_O_4_, stacked Co_3_O_4_, needled Co_3_O_4_ and floral Co_3_O_4_ have been synthesized, which show remarkably enhanced activity in the reduction of p-NP than commercial Co_3_O_4_ catalysts. Conical Co_3_O_4_ exhibits the highest catalytic activity because of the more reactive surface and more superior redox properties than other Co_3_O_4_ catalysts [34]. However, the morphologies of these Co_3_O_4_ catalysts are all microballs consisting of granules, sheets, nanoneedles or petals. The morphology, structure and physicochemical properties of different metal oxides vary greatly, and their catalytic performance can be markedly different. Therefore, it would be essential to investigate Co_3_O_4_ with different morphologies in order to explore the morphology–property–performance relationships and to provide insights into the design of Co_3_O_4_ catalysts to optimize their catalytic performance for p-NP reduction.

In this work, Co(OH)_2_ nanoplates, CoC_2_O_4_ nanorods and Co(OH)_2_ microflowers as catalyst precursors were synthesized by a simple precipitation method. CoC_2_O_4_ nanocubes were obtained by a facile hydrothermal method. Then, Co_3_O_4_ nanostructures with different morphologies were successfully constructed by calcining each precursor, and the as-prepared nanostructures retained the morphologies of the precursors. The composition, size, morphology and textural properties of the obtained Co_3_O_4_ nanostructures were characterized by various techniques, such as XRD, TEM, SEM, BET, TPR and XPS. Furthermore, the catalytic performance of Co_3_O_4_ with different morphologies was studied by monitoring the reduction of p-NP to p-AP in the presence of excess NaBH_4_. Then, the effect of the morphology of Co_3_O_4_ nanostructures on the catalytic performance was investigated, and a strong dependence was found.

## 2. Results and Discussion

### 2.1. Characterization of Co_3_O_4_ Nanostructures

#### 2.1.1. XRD

**Figure 1:**
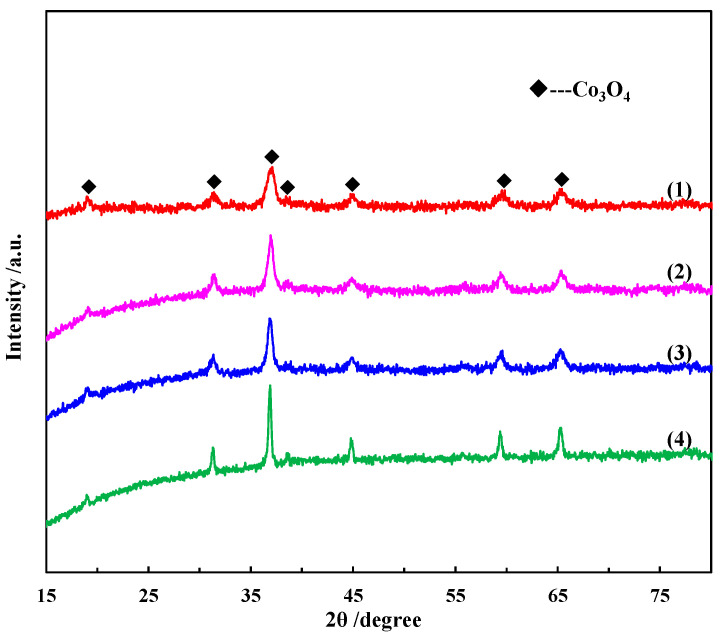
XRD patterns of Co_3_O_4_ nanostructures: (1) nanoplates, (2) microflowers, (3) nanorods and (4) nanocubes.

**Figure 2:**
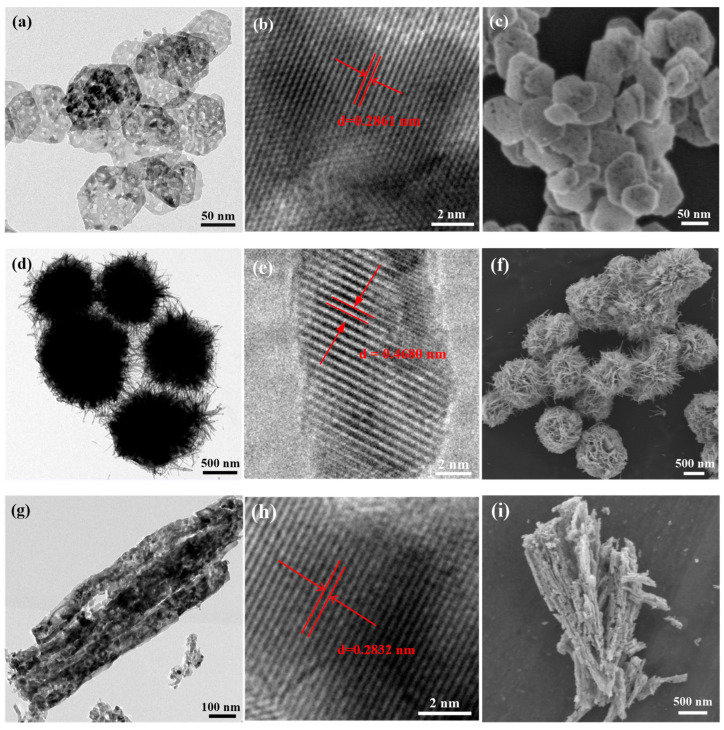

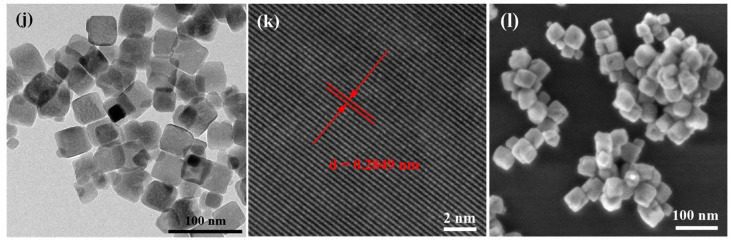
(**a**) TEM, (**b**) HRTEM and (**c**) SEM images of Co_3_O_4_ nanoplates; (**d**) TEM, (**e**) HRTEM and (**f**) SEM images of Co_3_O_4_ microflowers; (**g**) TEM, (**h**) HRTEM and (**i**) SEM images of Co_3_O_4_ nanorods; (**j**) TEM, (**k**) HRTEM and (**l**) SEM images of Co_3_O_4_ nanocubes.

Figure 1 shows the wide-angle XRD patterns of the as-prepared Co_3_O_4_ with different morphologies. The data used in the XRD pattern are all original data without background subtraction. These Co_3_O_4_ nanostructures were obtained from different precursors (the detailed materials and methods are described in Section 3). The morphology of these nanostructures is shown in Figure 2. The XRD peaks of the four samples match the JCPDS file (01-076-1802) of the cubic phase of Co_3_O_4_, indicating the complete transformation of the four precursors to Co_3_O_4_ via high-temperature calcination. Furthermore, the characteristic diffraction patterns of these Co_3_O_4_ nanostructures are identical except for their different intensities, implying that the same product was obtained but the crystallinity is different. For all the samples, the diffraction peaks at 19.03°, 31.32°, 36.90°, 38.61°, 55.75°, 59.45° and 65.34° in the XRD patterns of the Co_3_O_4_ nanostructures are well-matched with the (1 1 1), (2 2 0), (3 1 1), (2 2 2), (4 2 2), (5 1 1) and (4 4 0) facets of the cubic Co_3_O_4_, respectively. No peaks from the other phases or impurities were observed in the patterns of the Co_3_O_4_ nanostructures. In addition, there was an apparent increase of counts as the 2θ angle increased in the XRD patterns, especially for the Co_3_O_4_ nanocubes, nanorods and microflowers. This can be mainly caused by imperfect sample preparation or the sample not being aligned well during the scanning process.

#### 2.1.2. TEM and SEM

The morphology and structure of the as-prepared Co_3_O_4_ nanostructures were investigated by scanning electron microscopy (SEM) and transmission electron microscopy (TEM) (Figure 2). As shown in Figure 2a,c, the size distribution of the as-prepared Co_3_O_4_ nanoplates is nearly uniform and they all consist of well-defined hexagonal plates with an average side length of about 100 nm and an average thickness of about 20 nm. Numerous mesopores with a diameter of 5–10 nm were observed on the surface of the Co_3_O_4_ nanoplates, which were formed by the dehydration of Co(OH)_2_ during the high-temperature calcination process. No fragment was observed in the TEM and SEM images, indicating that all the particles were assembled into hexagonal nanoplates. The Co_3_O_4_ microflowers, assembled by a large number of intertwined nanorods and some nanosheets, have a uniform particle size distribution, and the diameter of the Co_3_O_4_ microflowers ranges from 0.8 µm to 1.2 µm. Since the intersection of nanorods can form various pores, there are many interstices in the microflowers. No cracked microcrystalline flower was observed in the SEM or TEM images. In addition to the flower-like structure, some of the nanorod pieces did not form a flower-like structure (Figure 2d,f). As shown in Figure 2g,i, the Co_3_O_4_ nanorod samples have an average length of about 1 μm and an average width of about 100 nm. Numerous pores and gullies were clearly observed on the surface of the nanorods, which can be attributed to the release of gas from CoC_2_O_4_ during the high-temperature decomposition process (described in Section 3.4). It is obvious from the images that some particles have not been assembled into nanorods. As shown in Figure 2j,l, the Co_3_O_4_ nanocube samples all have a typical cubic shape with some very small pores on the smooth surface and an average edge length of 40 nm. Furthermore, the size distribution of the Co_3_O_4_ nanocubes is regular, and no fragments were observed in the images. Figure 2b,e,h,k shows the HRTEM images of these Co_3_O_4_ nanostructures. The lattice fringes of the Co_3_O_4_ nanoplates, Co_3_O_4_ nanorods and Co_3_O_4_ nanocubes with an interplanar spacing between adjacent planes of 0.28–0.29 nm are attributed to the (2 2 0) facets of the cubic Co_3_O_4_ (JCPDS file value, 0.29 nm). The lattice space of the Co_3_O_4_ microflowers is 0.4680 nm, which is comparable to the (1 1 1) plane of Co_3_O_4_ (JCPDS file value, 0.467 nm) and, thus, is in good accordance with the XRD result.

#### 2.1.3. N_2_ Physisorption

**Figure 3:**
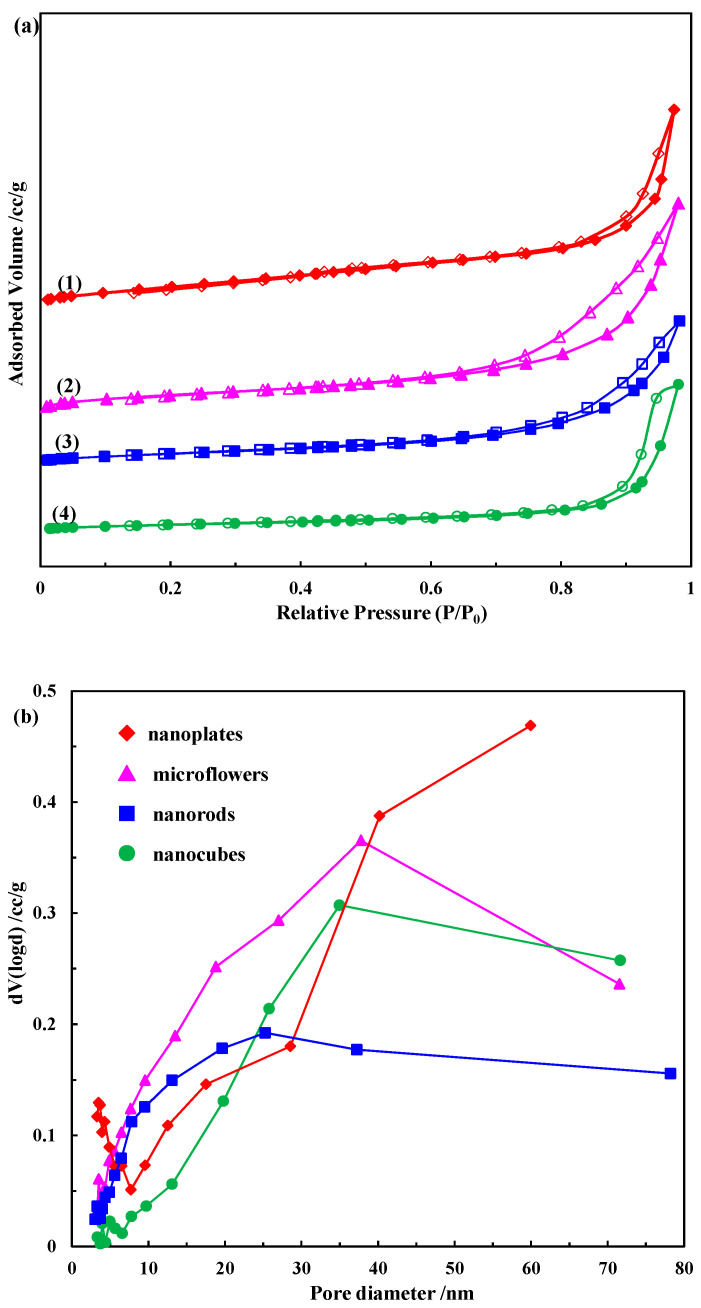
(**a**) N_2_ adsorption–desorption isotherm curves of Co_3_O_4_ nanostructures: (1) nanoplates, (2) microflowers, (3) nanorods and (4) nanocubes; (**b**) BJH pore size distributions of Co_3_O_4_ nanostructures.

In general, the catalytic activity of metal oxide nanomaterials is highly dependent on their surface area and pore volume; therefore, it is indispensable to investigate the relationship between the specific surface area and porosity of these Co_3_O_4_ nanostructures for a better elucidation of their catalytic activity. The nitrogen adsorption–desorption isotherms and BJH adsorption pore size distributions of these Co_3_O_4_ nanostructures with different morphologies are plotted in Figure 3. As depicted in Figure 3a, all the as-prepared Co_3_O_4_ samples exhibit a type IV adsorption isotherm pattern and an H1 hysteresis loop according to the IUPAC classification, demonstrating the presence of a regular mesoporous structure [35]. The specific surface areas and pore volumes of these nanostructures based on the BET results are summarized in Table 1. The Co_3_O_4_ nanoplates have the largest specific surface area of 106.3 m^2^·g^−1^. The specific surface areas of the Co_3_O_4_ microflowers, nanorods and nanocubes are 69.3 m^2^·g^−1^, 51.2 m^2^·g^−1^ and 31.7 m^2^·g^−1^, respectively. The total pore volumes of the Co_3_O_4_ nanoplates and microflowers are relatively similar, being 0.27 cc.g^−1^ and 0.30 cc.g^−1^, respectively. In contrast, the Co_3_O_4_ nanorods and nanocubes have identical pore volumes, but the value is relatively small at only 0.21 cc.g^−1^. The pore size distribution derived from the adsorption branch of the Co_3_O_4_ nanoplates shows a broad distribution, ranging from 3.3 nm to 59.9 nm, while those of the other three Co_3_O_4_ samples are even broader, ranging from 1.9 nm to 78.2 nm (Figure 3b), which can be attributed to the intra-aggregated pores within the agglomerated particles [36].

#### 2.1.4. H_2_-TPR

**Figure 4:**
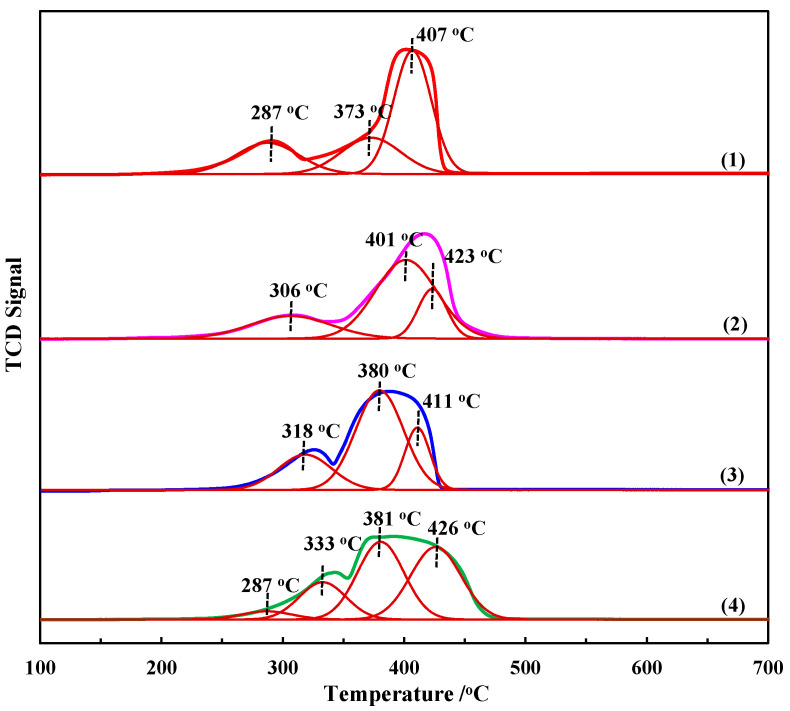
H_2_-TPR profiles and their deconvolution for Co_3_O_4_ nanostructures: (1) nanoplates, (2) microflowers, (3) nanorods and (4) nanocubes (measurement conditions: temperature from 100 °C to 700 °C at a rate of 10 °C/min using a stream of 10% H_2_/Ar balance with a flow rate of 40.18 cm^3^ STP/min).

The catalytic activity of a catalyst for redox reactions depends largely on its reducibility [37]. The H_2_-TPR profiles were determined to investigate the oxidation–reduction properties of the Co_3_O_4_ samples with different morphologies, as illustrated in Figure 4. All four Co_3_O_4_ nanostructures exhibit two distinct reduction peaks in the range of 200–500 °C, corresponding to an overall two-step reduction process with CoO as the intermediate (i.e., the reduction of Co^3+^ to Co^2+^ at a relatively low temperature and then Co^2+^ to Co at a relatively high temperature), similar to that reported in the literature [38]. Curve-fitting of the TPR profiles of the Co_3_O_4_ nanoplates, microflowers and nanorods resulted in the resolution of three reduction sub-processes, while four reduction sub-processes were identified in the TPR curve of the Co_3_O_4_ nanocubes. The peak located at a relative high temperature can be deconvoluted into two adjacent peaks, indicating that the reduction of Co^2+^ to Co over all four catalysts involved the overlapping of two events, and that Co^2+^ was not completely reduced to metallic Co in one step. The peaks of the Co_3_O_4_ nanoplates, microflowers and nanorods at a relatively low temperature can only be deconvoluted into one peak, indicating that Co^3+^ was reduced to Co^2+^ in one step. However, the peak of the Co_3_O_4_ nanocubes centered around such a low temperature can be fitted with two peaks at temperatures of 287 °C and 333 °C (area ratio 1:4.2). The weak peak at 287 °C may be attributed to the reduction of a small amount of Co^3+^ to Co^2+^ [39]. The relative area of the two peaks located at the low- and high-temperature regions can be determined by means of the deconvolution above. The area ratio of the two peaks is calculated to be 1:3.44 for the Co_3_O_4_ nanoplates, 1:3.40 for the Co_3_O_4_ microflowers, 1:3.37 for the Co_3_O_4_ nanorods and 1:3.37 for the Co_3_O_4_ nanocubes, which are all close to the stoichiometric ratio of Co^3+^ to Co^2+^ in Co_3_O_4_. Furthermore, all the Co_3_O_4_ samples were completely reduced to metallic cobalt before 500 °C. However, the peak position, peak intensity and half-peak width of these samples are different, suggesting that their redox properties are, to some extent, dependent on their morphologies. The first reduction peaks of the Co_3_O_4_ nanostructures are 287 °C for the Co_3_O_4_ nanoplates, 306 °C for the Co_3_O_4_ microflowers, 318 °C for the Co_3_O_4_ nanorods and 333 °C for the Co_3_O_4_ nanocubes. The results indicate that the reducibility of the Co_3_O_4_ nanoplates is the strongest among the four Co_3_O_4_ nanostructures, i.e., lattice oxygen species coordinated with Co^3+^ cations are more easily extracted from the surface of the Co_3_O_4_ nanoplates, thereby facilitating the electron transfer and the formation of more oxygen vacancies. This is followed by the Co_3_O_4_ microflowers. The decrease in reducibility of the Co_3_O_4_ nanorods and nanocubes can be attributed to the decrease in the specific surface areas, as observed from the BET analyses above [35].

#### 2.1.5. XPS

**Figure 5:**
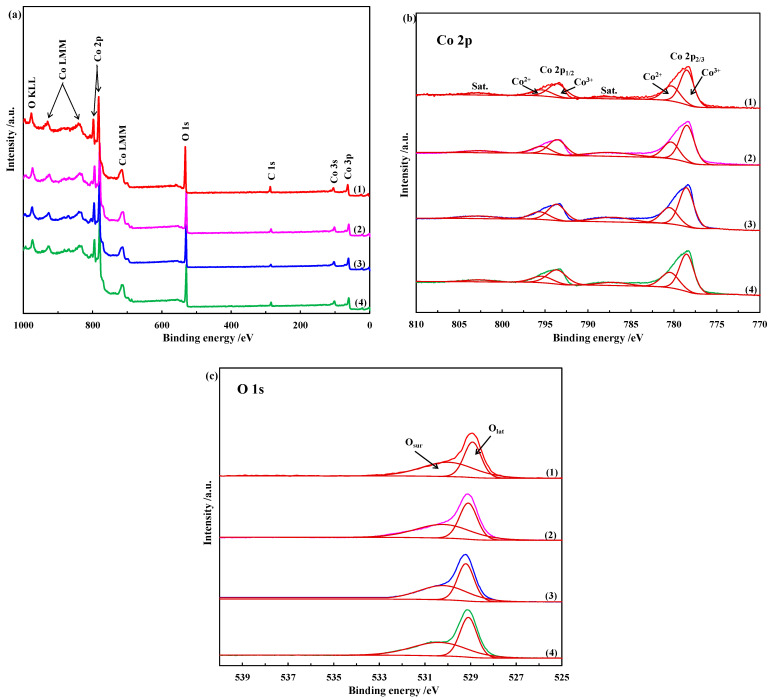
(**a**) XPS survey spectra; (**b**) Co 2p spectra; (**c**) O 1s spectra of (1) Co_3_O_4_ nanoplates, (2) Co_3_O_4_ microflowers, (3) Co_3_O_4_ nanorods and (4) Co_3_O_4_ nanocubes.

X-ray photoelectron spectroscopy was carried out to qualitatively characterize the chemical composition and surface chemical states of these Co_3_O_4_ nanostructures [40]. Figure 5a shows the XPS full spectra of these nanostructures. It is evident that each peak in the spectra can be attributed to Co, O or C elements, indicating the absence of other metallic or inorganic impurities. In general, the weak C1s peak can be attributed to the hydrocarbon contaminations inherent in the XPS analysis; therefore, these Co_3_O_4_ nanostructures are merely constituted of Co and O elements. The high-resolution XPS spectra of the Co element of the four Co_3_O_4_ samples are shown in Figure 5b to provide detailed information on the chemical oxidation states. The sharp peak at the binding energy of about 779.0 eV is characteristic of Co 2p_3/2_ with two curve-fitted peaks corresponding to Co^3+^ (778.5~778.55 eV) and Co^2+^ (780.4~780.55 eV). The shoulder peak with a binding energy of about 794.0 eV can be deconvoluted into two typical peaks of Co^3+^ (793.6~793.7 eV) and Co^2+^ (795.5~795.7 eV), which can be attributed to the corresponding Co 2p_1/2_. The shake-up satellite peaks with low intensity at ca. 8.8 eV from the main spin-orbit components of Co 2p_3/2_ are also characteristic of Co_3_O_4_. Apparently, the element Co is present in the chemical state of Co^2+^ and Co^3+^, and there is no metallic cobalt. The molar percentages of Co^2+^ and Co^3+^ in these Co_3_O_4_ nanostructures are obtained according to the integrated area ratios obtained by XPS splitting, as summarized in Table 2. Our previous work has demonstrated that when Co_3_O_4_ is treated with a NaBH_4_ aqueous solution, some of the Co^3+^ can be reduced to Co^2+^ and at the same time, oxygen vacancies are formed on the Co_3_O_4_ surface. More Co^2+^ on the surface of Co_3_O_4_ indicates that more Co^3+^ was reduced to Co^2+^ and more oxygen vacancies were formed [16]. Therefore, the amount of oxygen vacancies on the surface of the Co_3_O_4_ nanostructures is ordered as follows: Co_3_O_4_ nanoplates > Co_3_O_4_ nanorods > Co_3_O_4_ microflowers > Co_3_O_4_ nanocubes. Furthermore, the high-resolution O 1s spectra of these Co_3_O_4_ nanostructures observed at 528.9~529.2 eV can be deconvoluted into two typical peaks with binding energies of 528.9~529.2 eV and 530.0~530.4 eV, which are attributed to the lattice oxygen (O_lat_) and the surface adsorption oxygen species (O_sur_), respectively [41]. No characteristic peaks assigned to oxygen vacancies were observed, indicating that the amount of oxygen vacancies formed was very small.

### 2.2. Catalytic Performance of Co_3_O_4_ Nanostructures

The catalytic reduction of p-NP to p-AP in the presence of excess NaBH_4_ was chosen as a probe reaction to evaluate the catalytic performance of Co_3_O_4_ nanostructures with different morphologies. The reaction is considered as a thermodynamically favorable process according to the standard electrode potential (p-NP/p-AP = −0.76 V, H_3_BO_3_/BH_4_^−^ = −1.33 V), but it is difficult for electrons to transfer from BH_4_^−^ to the p-NP substrate due to the large difference in the redox potential and the high kinetic barrier between the mutually exclusive negative donor (BH_4_^−^ ions) and acceptor (p-nitrophenolate ions) [42]. Therefore, the NaBH_4_ solution itself cannot reduce p-NP to p-AP in the absence of a highly efficient catalyst. Co_3_O_4_ has been proven as an effective catalyst for the reduction of p-NP, which can reduce the kinetic barrier [43].

**Figure 6:**
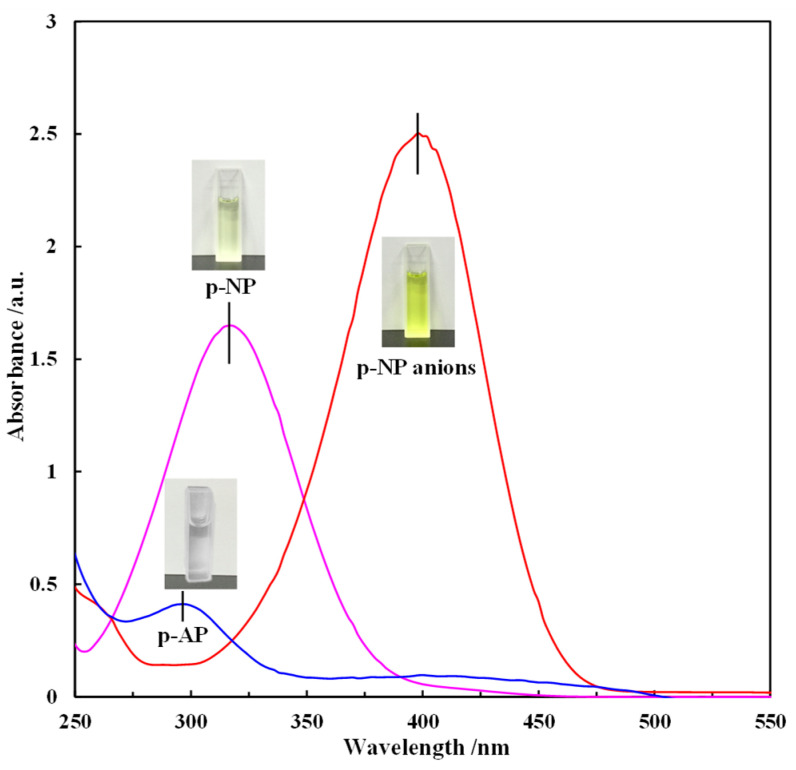
UV-vis absorption spectra of p-NP, p-NP anions and p-AP.

The catalytic reduction of p-NP to p-AP using Co_3_O_4_ nanostructures as a catalyst was conducted in a standard quartz cell and monitored by an in situ UV-vis spectrophotometer. The p-NP solution was first homogeneously mixed with a NaBH_4_ solution, and then different shapes of Co_3_O_4_ nanostructures were added to initiate the reduction reaction. The pale-yellow aqueous solution of p-NP exhibited a strong absorption peak centered at 317 nm, as shown in Figure 6. When the NaBH_4_ aqueous solution was added, the absorption peak was red-shifted to 400 nm, accompanied by a color change to green-yellow, which could be attributed to the formation of p-nitrophenolate ions (i.e., p-NP anions) under alkaline conditions [44]. When the Co_3_O_4_ nanostructures were introduced into the mixed solution of p-NP and NaBH_4_, the absorption peak of p-NP at 400 nm gradually decreased with the fading of the yellow color and the simultaneous appearance of a new absorption peak at 300 nm (which can be attributed to the formation of p-AP). The absorption peak at 400 nm was significantly stronger than that at 300 nm; therefore, the progress or kinetics of the reaction was measured based on the concentration of p-NP anions in the reaction mixture by monitoring the change in the absorbance at 400 nm at different time intervals.

#### 2.2.1. Catalytic Activity Test

**Figure 7:**
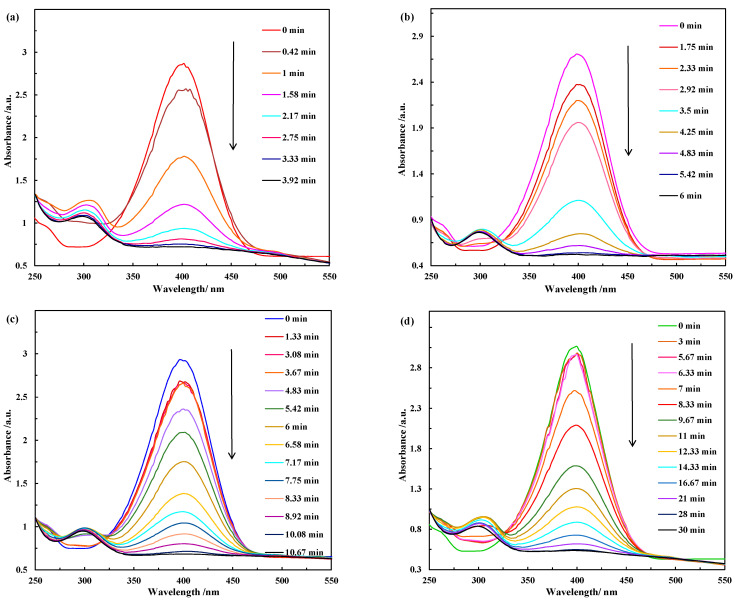
The evolution of UV-vis absorption spectra during the catalytic reduction of p-NP in the presence of different catalysts: (**a**) Co_3_O_4_ nanoplates, (**b**) Co_3_O_4_ microflowers, (**c**) Co_3_O_4_ nanorods, (**d**) Co_3_O_4_ nanocubes. C_p-NP_ = 0.125 mmol/L, NaBH_4_/p-NP = 100, m_cat_ = 0.2 mg.

Figure 7 shows the extinction UV-vis absorption spectra of p-NP after reaction for different durations in the presence of 0.2 mg of Co_3_O_4_ nanostructures with different morphologies. It can be seen that the absorption peak of p-NP at 400 nm decreased with time and a new peak was simultaneously generated at a wavelength of 300 nm, indicating the reduction of p-NP to p-AP. Figure 7a–d shows that with the prolongation of the reaction, the decrease rate of the peak intensity at 400 nm varies greatly among these Co_3_O_4_ nanostructures, implying that different shapes of Co_3_O_4_ have different catalytic activities for the p-NP reduction reaction. When the Co_3_O_4_ nanoplates were used as catalyst, the time to complete the reduction process was less than 3.9 min (Figure 7a). The complete conversion of p-NP to p-AP in the presence of Co_3_O_4_ microflowers took 5.4–6.0 min (Figure 7b). These are in comparison with 10.7 min to complete the reaction over Co_3_O_4_ nanorods (Figure 7c) and approximately 30.0 min over Co_3_O_4_ nanocubes (Figure 7d). Thus, Co_3_O_4_ nanoplates render the best catalytic activity, followed by Co_3_O_4_ microflowers, then nanorods and, finally, nanocubes.

In addition to the different time required for the complete conversion of p-NP to p-AP over these Co_3_O_4_ nanostructures, an induction time (*t*_0_) is present, during which no reaction occurred initially. The induction time of these Co_3_O_4_ nanostructures differs significantly, as summarized in Table 3. The order of induction time required for the reduction of p-NP catalyzed by these Co_3_O_4_ catalysts is as follows: nanoplates < microflowers < nanorods < nanocubes, which is consistent with the order of the catalytic activity, that is, the shorter the induction time, the higher the catalytic activity. There have been many reports on the existence and happenstance of the induction time. Three main hypotheses have been found in the literature, namely, the spontaneous reconstruction time of the catalyst surface, the activation time of the catalyst active site, and the time required for reactants to diffuse onto the catalyst surface; however, the existence of the induction time is still controversial, and a concrete reason needs to be further verified [45].

**Figure 8:**
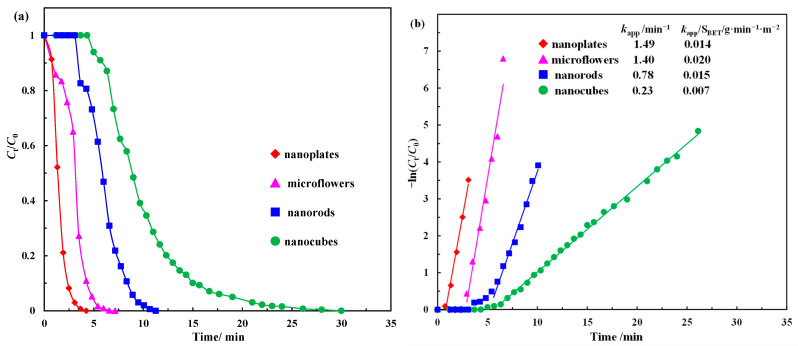
(**a**) *C_t_*/*C*_0_ as a function of reaction time; (**b**) kinetic analysis over different Co_3_O_4_ nanostructure catalysts reduced by aqueous NaBH_4_. C_p-NP_ = 0.125 mmol/L, p-NP/NaBH_4_ = 100 (molar ratio), m_cat_ = 0.2 mg (catalyst weight).

As a typical model reaction, the kinetics of the catalytic reduction of p-NP to p-AP by NaBH_4_ has been extensively investigated. In this work, the initial molar ratio of NaBH_4_ to p-NP was 100, i.e., there was a significant excess of NaBH_4_. In this case, the concentration of BH_4_^−^ remained essentially constant throughout the reaction, and the reaction rate was independent of the concentration of NaBH_4_. Then, it is reasonable to assume that the reaction follows the pseudo-first-order kinetics with respect to p-NP. The kinetic equation for the reduction reaction can be described as follows:

−*ln*(*C_t_*/*C*_0_) = −*ln*(*A_t_*/*A*_0_) = *k*_app_*t* (1)

where *A*_t_ and *A*_0_ are the absorbance values of p-NP (λ = 400 nm) at time *t* and at the initial time, respectively. *C*_t_ and *C*_0_ represent the equivalent concentrations of the p-nitrophenolate ions obtained from *A*_t_ and *A*_0_, respectively. *K*_app_ and *t* represent the apparent rate constant and reaction time, respectively. Therefore, the apparent rate constant values of these Co_3_O_4_ nanostructures are determined by the slopes of the respective linear curves of *t* versus −ln(*A_t_*/*A*_0_). As shown in Figure 8, the apparent rate constant values for the p-NP reduction reaction in the presence of 0.2 mg of different shapes of Co_3_O_4_ are 1.49 min^−1^ (nanoplates), 1.40 min^−1^ (microflowers), 0.78 min^−1^ (nanorods) and 0.23 min^−1^ (nanocubes). The activity sequence for the reduction of p-NP catalyzed by Co_3_O_4_ with different morphologies is as follows: Co_3_O_4_ nanoplates > Co_3_O_4_ microflowers > Co_3_O_4_ nanorods > Co_3_O_4_ nanocubes.

The literature has demonstrated that the reduction of p-NP to p-AP is a surface-controlled process that can be analyzed by the Langmuir–Hinshelwood mechanism [35]. The model reaction assumes that p-NP ions and BH_4_^−^ are competitively adsorbed on the surface of Co_3_O_4_. The adsorbed BH_4_^−^ reacts with the Co_3_O_4_ surface to transfer active hydrogen species and electrons to the surface. These Co_3_O_4_ catalysts with different morphologies then serve as catalysts to relay active hydrogen species and electrons to the adsorbed p-NP anions to form p-AP, which is considered as the rate-determining step. The generated p-AP is then desorbed from the catalyst surface to expose the active sites for the next reduction cycle. The adsorption and desorption processes are supposed to be fast; therefore, the efficient adsorption of p-NP and BH_4_^−^ and the efficient desorption of p-AP, as well as the superior electron transport capacity are key factors for the excellent activity of a catalyst [46]. It is well known that the surface properties of the catalyst can be tailored by controlling the morphology, size, composition and porosity of the catalyst, thereby improving its catalytic performance [47,48]. Different morphologies of Co_3_O_4_ are associated with different exposed crystalline planes, specific surface area, surface defects, pore structure and reducibility, which can, in turn, have a significant impact on their catalytic performance [49].

In general, the large specific surface area of the catalyst is conducive to the adsorption of reactant molecules (i.e., BH_4_^−^ and p-NP) on its surface, and its surface can expose more active sites, which largely ensures its excellent catalytic performance. According to the BET results in Figure 3, the specific surface areas of the Co_3_O_4_ nanoplates, microflowers, nanorods and nanocubes are 106.3, 69.3, 51.2 and 31.7 m^2^/g, respectively, which is consistent with the order of their catalytic activity [33]. Pore structure is also one of the most important factors affecting catalyst performance, as the abundance of interparticle mesopores and high pore volume can provide more active surface sites for a heterogeneous catalytic reaction. It can be clearly seen from the TEM and SEM results (Figure 2) that there are many small pores on the surface of the Co_3_O_4_ nanoplates, and the surface of the Co_3_O_4_ microflowers is rough. The BET results (Figure 3 and Table 1) show that the total pore volumes of the Co_3_O_4_ nanoplates and microflowers are 0.27 and 0.30 cc/g, respectively, which is significantly higher than those of the Co_3_O_4_ nanorods and nanocubes. Despite the rough surface of the Co_3_O_4_ nanorods, the total pore volume is only 0.21 cc/g, which is the same as that of the Co_3_O_4_ nanocubes with a smooth surface. As a result, the catalytic activity of the Co_3_O_4_ nanoplates and microflowers is much higher than that of the Co_3_O_4_ nanorods and nanocubes [50]. Oxygen vacancies, as a common inherent surface defect in metal oxides, have been proven to significantly affect or even modify the physical and chemical properties of materials and are an effective means to regulate the structure and catalytic performance of catalysts. In addition, our previous results have demonstrated that oxygen vacancies can act as active sites for the reduction of p-NP [16]. The XPS results show that the amount of oxygen vacancies on the surface of Co_3_O_4_ nanostructures is ordered as follows: Co_3_O_4_ nanoplates > Co_3_O_4_ nanorods > Co_3_O_4_ microflowers > Co_3_O_4_ nanocubes. Therefore, the catalytic activity of the Co_3_O_4_ nanoplates is the highest, and that of the Co_3_O_4_ nanocubes is the lowest. For the reduction of p-NP, Co_3_O_4_ served as a catalyst to transfer active hydrogen species and electrons from the donor BH_4_^−^ to the acceptor p-NP. Therefore, the reducibility of Co_3_O_4_ also greatly affects its catalytic performance. According to the TPR results (Figure 4), the order of the reducibility of these different Co_3_O_4_ catalysts is as follows: Co_3_O_4_ nanoplates > Co_3_O_4_ microflowers > Co_3_O_4_ nanorods > Co_3_O_4_ nanocubes, which is consistent with the order of their catalytic activity. In summary, different morphologies of Co_3_O_4_ render different properties, such as different specific surface area, surface defects, porosity and reducibility, which, in turn, significantly affect their catalytic performance.

Among these factors, the specific surface area (S_BET_) seems to have the greatest influence on the catalytic performance, given that it is much higher for the Co_3_O_4_ nanoplates than that for the other shapes (Table 1). To better elucidate the role of other factors, the reaction rate constants per unit specific surface area (*k*_app_/S_BET_) were adopted to compare the activity of Co_3_O_4_ with different morphologies. The values of *k*_app_/S_BET_ are 0.020 g·min^−1^·m^−2^ for the Co_3_O_4_ microflowers, 0.015 g·min^−1^·m^−2^ for the Co_3_O_4_ nanorods, 0.014 g·min^−1^·m^−2^ for the Co_3_O_4_ nanoplates and 0.007 g·min^−1^·m^−2^ for the Co_3_O_4_ nanocubes (Figure 8b, Table 4). The sequence of the catalytic activity per unit-specific surface area of these Co_3_O_4_ nanostructures is as follows: Co_3_O_4_ microflowers > Co_3_O_4_ nanorods > Co_3_O_4_ nanoplates > Co_3_O_4_ nanocubes. The Co_3_O_4_ microflowers exhibited the best catalytic activity per unit specific surface area, which can be attributed to its largest total pore volume. Therefore, compared with the specific surface area and total pore volume, the effect of reducibility and surface defects is believed to be relatively small in determining the current catalyst performance.

#### 2.2.2. Stability Test

**Figure 9:**
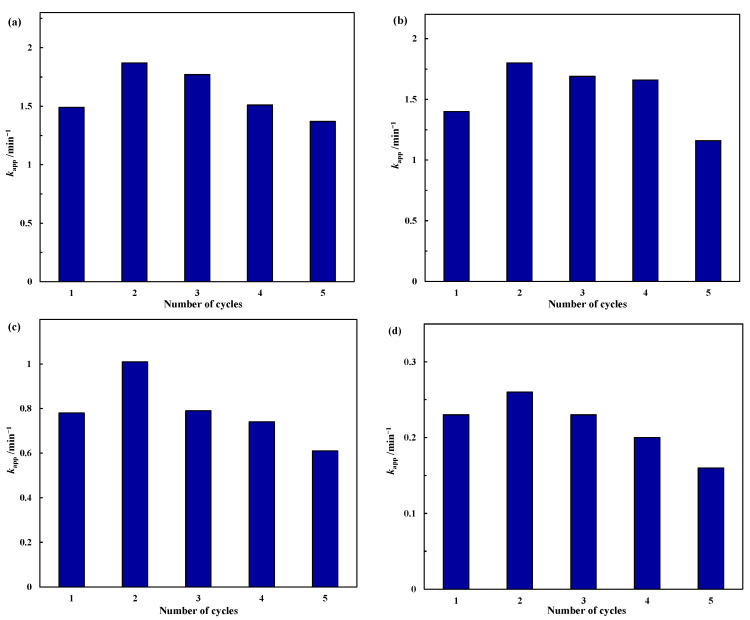
Recyclability of Co_3_O_4_ nanostructures for the catalytic reduction of p-NP to p-AP by excess NaBH_4_: (**a**) Co_3_O_4_ nanoplates; (**b**) Co_3_O_4_ microflowers; (**c**) Co_3_O_4_ nanorods; (**d**) Co_3_O_4_ nanocubes. C_p-NP_ = 0.125 mmol/L, p-NP/NaBH_4_ = 100, m_cat_ = 0.2 mg.

Considering the economic benefits and environmental factors in the practical applications, recyclability is another criterion to evaluate the performance of catalysts in addition to their catalytic activity. The recyclability of these Co_3_O_4_ nanostructures was evaluated by continuously adding a concentrated p-NP aqueous solution after each cycle to adjust the concentration of p-NP to its initial value of 0.125 mmol/L before the reaction. The NaBH_4_ concentration in the first cycle was 12.5 mmol/L, which is 100 times the concentration of p-NP, and no NaBH_4_ was added in the subsequent cycles. As can be seen from Figure 9, although the apparent rate constants of all the Co_3_O_4_ nanostructures are somewhat reduced, they show no significant deactivation and still maintain satisfactory stability over multiple consecutive cycles. After five consecutive cycles, the apparent rate constants could still reach 1.37 min^−1^ for the Co_3_O_4_ nanoplates, 1.16 min^−1^ for the Co_3_O_4_ microflowers, 0.61 min^−1^ for the Co_3_O_4_ nanorods and 0.16 min^−1^ for the Co_3_O_4_ nanocubes. To better evaluate the performance of the current catalysts, the activity of some common (supported) noble metal catalysts and other cobalt-based catalysts are summarized in Table 4. As can be seen from the table, the catalytic activity (*k*_nor_) of the as-prepared Co_3_O_4_ nanoplates and Co_3_O_4_ microflowers in this work is comparable or even better than that of some noble metal catalysts and supported ones (e.g., Au, Pd, Au-Cu_x_O_y_ in HPSNs and Ag-CeO_2_) and is significantly higher than that of other cobalt-based catalysts (e.g., PdO-Co_3_O_4_, NiCo_2_O_4_, and Co_3_O_4_@C). The results here clearly demonstrate the relatively high efficiency of the Co_3_O_4_ nanoplates and microflowers for the catalytic reduction of p-NP.

## 3. Materials and Methods

### 3.1. Materials

Cobalt nitrate hexahydrate (Co(NO_3_)_2_·6H_2_O), cobalt acetate tetrahydrate (Co(CH_3_COO)_2_·4H_2_O), cobalt chloride hexahydrate (CoCl_2_·6H_2_O), sodium hydroxide (NaOH), urea (CO(NH_2_)_2_), oxalic acid (H_2_C_2_O_4_), polyvinyl pyrrolidone K90 ((C_6_H_9_NO)_n_, PVP), sodium dodecyl benzene sulfonate (C_18_H_29_NaO_3_S, SDBS), sodium borohydride (NaBH_4_) and p-nitrophenol (C_6_H_5_NO_3_) of analytical grade were purchased from Aladdin Industrial Corporation (Shanghai, China). All the chemicals were used as received without further treatment. Deionized water was used in all the experiments.

### 3.2. Preparation of Co(OH)_2_ Nanoplates

The Co(OH)_2_ nanoplates were synthesized according to our previous work [16]. Briefly, CoCl_2_·6H_2_O (5 mmol) and NaOH (50 mmol) were dissolved separately in 50 mL deionized water. The precipitation was started by the dropwise addition of CoCl_2_ and NaOH aqueous solutions into a three-necked flask containing 50 mL deionized water at 50 °C under N_2_ atmosphere. The obtained pink suspension was stirred at 50 °C for 1 h. The N_2_ flow was then stopped. The product was cooled to room temperature, centrifuged, washed several times with deionized water and dried in the oven (60 °C) for 8 h.

### 3.3. Preparation of Co(OH)_2_ Microflowers

The Co(OH)_2_ microflowers were synthesized by a simple precipitation method. In a typical experiment, Co(NO_3_)_2_·6H_2_O (4 mmol) and CO(NH_2_)_2_ (0.5 mol) were dissolved in 50 mL deionized water. Then, polyvinyl pyrrolidone (3.85 µmol) was added to the solution described above. The mixture was ultrasonicated for 1 h to form a homogeneous solution. The solution was then transferred to a three-necked flask and heated in a water bath at 80 °C for 6 h with magnetic stirring. The flask was semi-sealed during the reaction process. Finally, a purple precipitate was obtained. After cooling to room temperature, the purple precipitate was centrifuged, washed several times with deionized water and dried in the oven (60 °C) for 8 h.

### 3.4. Preparation of CoC_2_O_4_ Nanorods

The CoC_2_O_4_ nanorods were synthesized by a simple precipitation method. Briefly, Co(CH_3_COO)_2_·4H_2_O (5.9 mmol) and NaOH (10 mmol) were dissolved into 50 mL and 10 mL deionized water, respectively. Then, 0.6 g of H_2_C_2_O_4_ was added to the Co(CH_3_COO)_2_ aqueous solution. The precipitation process was started by the dropwise addition of a NaOH aqueous solution into the Co(CH_3_COO)_2_ aqueous solution under continuous stirring. The obtained pink suspension was stirred at 85 °C for 12 min. The product was cooled to room temperature, centrifuged, washed several times with deionized water and dried in the oven (60 °C) for 8 h.

### 3.5. Preparation of CoC_2_O_4_ Nanocubes

The CoC_2_O_4_ nanocubes were synthesized by a facile hydrothermal method. In a typical synthesis, 4 mmol Co(CH_3_COO)_2_·4H_2_O and 5 mmol NaOH were dissolved in 20 mL deionized water, respectively. The precipitation was initiated at room temperature by the dropwise addition of a NaOH aqueous solution to the Co(CH_3_COO)_2_ aqueous solution with continuous stirring. The obtained suspension was stirred for another 0.5 h at room temperature. Then, 1.2 g SDBS was added to the suspension described above. The suspension was transferred to a 100 mL Teflon-lined stainless-steel autoclave, and then the autoclave was maintained at 160 °C for 36 h. The temperature was programmed to increase from room temperature to 160 °C at a heating rate of 2.0 °C/min. After cooling to room temperature, the black precipitate was centrifuged, washed several times with deionized water and dried in the oven (60 °C) for 8 h.

### 3.6. Preparation of Reduced Co_3_O_4_

The as-prepared Co(OH)_2_ nanoplates, Co(OH)_2_ microflowers, CoC_2_O_4_ nanorods and CoC_2_O_4_ nanocubes were calcined at 300 °C for 2 h in air to obtain the corresponding Co_3_O_4_ nanostructures. The temperature was programmed to increase from room temperature to 300 °C at a ramp of 2 °C/min. Then, these Co_3_O_4_ nanostructures were separately soaked in a NaBH_4_ aqueous solution (0.05 mol/L) at 30 °C for 40 min. Afterwards, the powder was collected via centrifugation, washed several times with deionized water and used directly for the catalytic reduction of p-NP to p-AP.

### 3.7. Catalyst Characterization

The powder X-ray diffraction (XRD) analyses of the as-prepared samples at room temperature were recorded by a PANalytical X’pert-Pro powder X-ray diffractometer equipped with Cu Ka monochromatic radiation (λ = 0.1541 nm) with 2θ ranging from 10° to 90° at a scanning rate of 5°/min. The X-ray tube was operated under 40 kV and 40 mA. Nitrogen sorption measurements of the samples were performed on a Quadrasorb SI adsorption analyzer. The specific surface areas and pore properties were calculated from the adsorption–desorption isotherms collected at 77 K. Transmission electron microscopy (TEM) were taken on a JEOL JEM-2100 at an accelerating voltage of 120 kV, and scanning electron microscopy (SEM) were taken on a JEOL JSM-7800F at an accelerating voltage of 3 kV to examine the morphologies of the as-prepared samples. Temperature-programmed reduction (H_2_-TPR) was carried out on a Micromeritics AutoChem 2920 apparatus. A 10% mixture of H_2_/Ar passed through the catalyst bed at a flow rate of 40 mL min^−1^, and at the same time, the temperature was programmed to increase from 50 °C to 700 °C at a heating rate of 10 °C/min.

### 3.8. Catalytic Reduction of p-NP to p-AP by Excess NaBH_4_

The catalytic reduction of p-NP to p-AP was conducted under controllable conditions to evaluate the catalytic performance of the obtained nanostructures. Typically, the reduction reaction was conducted in a 3.5 mL standard quartz cell at room temperature (ca. 23 °C). Excess NaBH_4_ aqueous solution was chosen as the reducing agent. Approximately 2.0 mL p-NP aqueous solution (0.175 mmol/L) was first mixed with 0.7 mL freshly prepared NaBH_4_ solution (0.05 mol/L) in the quartz cell. Then, 0.1 mL suspension containing one of the obtained Co_3_O_4_ nanostructures (2 g/L) was quickly injected into the mixture under temperature control and rapid stirring. The reduction process was monitored by detecting the extinction of p-NP via an in situ UV-vis spectrophotometer (METASH UV8000) over a scanning range of 200 nm–600 nm. The kinetic evaluation was judged by the disappearance of the p-NP adsorption peak at 400 nm as a function of the reaction time.

## 4. Conclusions

Co_3_O_4_ nanoplates, microflowers, nanorods and nanocubes were successfully synthesized by facile precipitation or hydrothermal methods, followed by a thermal decomposition process. The catalytic performance of these Co_3_O_4_ nanostructures for the reduction of p-NP to p-AP in the presence of excess NaBH_4_ was studied. The order of the catalytic activity of these Co_3_O_4_ nanostructures is nanoplates > microflowers > nanorods > nanocubes. Co_3_O_4_ nanoplates exhibit the highest catalytic activity due to their largest specific surface area, relatively high total pore volume, abundant oxygen vacancies and strong reducibility. The results indicate that Co_3_O_4_ catalysts with different morphologies exhibit different properties, such as different specific surface area, porosity, surface defects and reducibility, which, in turn, significantly affect their catalytic performance. Therefore, morphology control in the preparation of Co_3_O_4_ nanocrystals is crucial for the preparation of highly efficient heterogeneous catalysts. The controllable synthesis of Co_3_O_4_ nanostructures with different morphologies is expected to bring new opportunities for regulating the catalytic performance of metal oxide catalysts.

## Figures and Tables

**Table 1:** Surface properties of Co_3_O_4_ nanostructures.

Catalyst	BET Surface Area (m^2^/g)	Total Pore Volume (cc/g)	Average Pore Diameter (nm)
Nanoplates	106.3	0.27	3.5
Microflowers	69.3	0.30	3.5
Nanorods	51.2	0.21	7.8
Nanocubes	31.7	0.21	3.5

**Table 2:** The molar percentages of Co^2+^ and Co^3+^ in Co_3_O_4_ nanostructures.

Catalyst	Co^2+^ (mol%)	Co^3+^ (mol%)	Co^3+^/Co^2+^
Nanoplates	34.1	65.9	1.93
Microflowers	33.8	66.2	1.96
Nanorods	33.9	66.1	1.95
Nanocubes	33.5	66.5	1.99

**Table 3:** The induction times of various Co_3_O_4_ nanostructures for the reduction of p-NP ^a^.

Catalyst	*t*_0_/min
Nanoplates	*t*_0_ < 0.42
Microflowers	*t*_0_ < 1.75
Nanorods	4.25 < *t*_0_ < 4.83
Nanocubes	6.33 < *t*_0_ < 7

^a^ Based on the reaction results in Figure 7 and Figure 8.

**Table 4:** Comparison of the rate constants of different catalysts for the catalytic reduction of p-NP to p-AP by NaBH_4_ in the recent literature and this work.

Catalyst	m_cat_ ^a^	*k*_app_	*k*_nor_ ^b^	S_BET_	*k*_app_/S_BET_	Reference
	g/L	min^−1^	L·min^−1^·g^−1^	m^2^/g	g·min^−1^·m^−2^	
Au	0.0034	0.1540	44.814	-	-	[51]
Pd	0.0462	0.7300	15.801	-	-	[52]
Hg/Pd	1.4286	3.5040	2.453	-	-	[53]
Au-Cu_x_O_y_ in HPSNs	0.2500	0.9600	3.840	86.7	0.0111	[54]
Au/TNT	0.3750	0.0610	0.163	124.0	0.0005	[55]
PdCu-LDHs	0.3107	1.1000	3.540	-	-	[56]
Ag-CeO_2_	0.1200	0.6560	5.467	5.6	0.1171	[57]
Co/Eatp@C	0.0500	0.6900	13.80	236.5	0.0029	[58]
PdO-Co_3_O_4_	0.8333	1.3100	1.572	67.3	0.0195	[59]
Co_3_O_4_@C	1.6667	0.7550	0.453	5.1	0.1480	[60]
NiCo_2_O_4_	0.5000	0.1260	0.252	68.4	0.0018	[61]
Co_3_O_4_ nanoplates	0.0714	1.4900	20.868	106.3	0.0140	This work
Co_3_O_4_ microflowers	0.0714	1.4000	19.600	69.3	0.0202	This work
Co_3_O_4_ nanorods	0.0714	0.7800	10.920	51.2	0.0152	This work
Co_3_O_4_ nanocubes	0.0714	0.2300	3.221	31.7	0.0073	This work

^a^ The mass concentration of the catalyst in the solution. ^b^ The mass-normalized rate constant calculated based on the catalyst amount, including the support based on the equation: *k*_nor_ = *k*_app_/m_cat_.

## Data Availability

All the data are available in the manuscript.
